# A new site for *Luticola vanheurckii* Van de Vijver & Levkov (Bacillariophyta) – the second record from Europe

**DOI:** 10.2478/s11756-018-0161-z

**Published:** 2018-12-13

**Authors:** Teresa Noga, Mateusz Rybak

**Affiliations:** 1 Department of Soil Studies, Environmental Chemistry and Hydrology, Faculty of Biology and Agriculture, University of Rzeszów, 35-601 Rzeszów, Poland; 2 Department of Agroecology, Faculty of Biology and Agriculture, University of Rzeszów, 35-601 Rzeszów, Poland

**Keywords:** The genus *Luticola*, Distribution, New record, Taxonomy, Ecology

## Abstract

This paper presents the first record of *Luticola vanheurckii* Van de Vijver & Levkov, outside of the type locality (second record in the Europe). Authors present habitat characteristic of the species, with LM and SEM micrographs. *Luticola vanheurckii* was found in a small outflow of water from storm sewers in Stalowa Wola (SE Poland). *Luticola vanheurckii* occurred only rarely (0–5 valves per slide), in a small, shallow puddle which was supplied by the water from the outflow. This species was recorded in sediments and on plants submerged in water with a wide range of conductivity (347–22,100 μS cm^−1^), chloride content (64.56–6195.0 mg l^−1^) and sodium ions (43.04–3789.1 mg l^−1^). The cells observed in the examined material were slightly longer and wider than those from type locality.

## Introduction

*Luticola* is a large, widespread genus with around 200 different species. Originally, species of the genus *Luticola* were included in the genus *Navicula*, but due to differences in nearly every aspect of the morphology and cell organization, they were displaced into a new genus, with the type species is *Luticola mutica* D.G. Mann. *Luticola* grow especially frequent in freshwater or slightly salty waters and in estuaries, on soils and mosses or other subaerial habitats (Round et al. 1990; Hofmann et al. 2011; Bąk et al. 2012; Levkov et al. 2013).

Several occurrences of *Luticola* species came from territory of Poland (e.g., Rakowska 2001; Siemińska and Wołowski 2003; Wojtal 2009; Bąk et al. 2012). Thirteen taxa of this genus have been identified so far within the Subcarpathian province (Noga et al. 2014; Noga et al. 2017).

*Luticola vanheurckii* Van de Vijver & Levkov, species described from Belgium and known only from the type locality (Levkov et al. 2013). In this studies was recorded in south-eastern Poland. Species descriptions included LM and SEM documentation, ecological characteristics and discussion on its distribution are presented.

## Study area

**Fig. 1:**
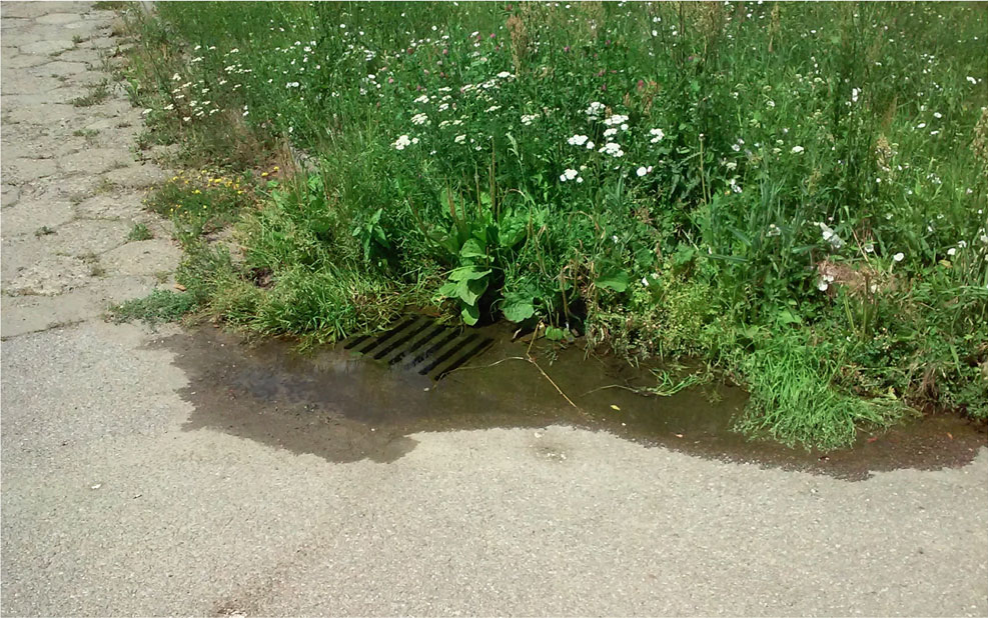
Sampling site – outflow from the storm water drainage, date: 15.06.2016, photo by M. Rybak

The study was performed on a small outflow of water in Stalowa Wola, south-eastern Poland (50°34'17.67"N, 22°04'33.78"E), which started in the storm sewage. During material sampling in March and June 2016, the water flowed on the surface of the asphalt road ca. 250 m (Fig. 1) and drifted into a small puddle, on the sunny side of the road. At this time puddle was not dried out, but depends from precipitation level, the size of puddle was change. From November 2016, the water stopped flowing from the storm sewage, and for this reason the material was collected from the puddle only. The puddle was shallow, from a few millimeters to about 10 cm.

## Material and methods

In March and June 2016 samples were collected from a puddle and water outflow. In November 2016, January and March 2017, material was taken only from a puddle. Algal mats developed on the surface of the road and parts of plants submerged in water were taken.

Water temperature, pH and electrolytic conductivity were measured in situ. A detailed water chemical analysis was performed in a laboratory using a DIONEX ICS–5000 + DC Thermo scientific ion chromatograph. In March 2016 samples were collected for first time, and only for diatom assemblage study, therefore no chemical analysis were perform.

Diatom samples were collected and prepared according to Kawecka (1980). Part of each sample was cleaned with a chromic cleaning mixture (mixture of sulphuric acid and potassium dichromate). After complete dissolution of organic matter samples were washed in a centrifuge (at 2500 rpm) several times, until the chromic mixture had been removed. Diatoms were mounted in Pleurax resin (refractive index 1.75) and identified and counted under a Carl Zeiss Axio Imager A2 light microscope (LM) under 1000× magnification, with differential interference contrast (DIC) for oil immersion. For scanning electron microscope (SEM) observations, samples were coated with 20 nm of gold using a Quorum Q 150OT ES Turbo-Pumped Sputter Coater and observed under a Hitachi SU 8010. Ecological characterization and identification of diatoms were performed according to Lange-Bertalot (2001), Hofmann et al. (2011), Levkov et al. (2013) and Lange-Bertalot et al. (2017). Species composition was determined by counting 300 valves on randomly selected fields of view under LM. Species with a content above 5% of all counted valves in diatom assemblage were defined as the most abundant.

## Results

The water of the studied puddle was alkaline, the highest pH (9.4) was measured in June 2016, when the water flowed on the asphalt. During this time, water was characterized by average conductivity values (347 μS cm^−1^) and high nitrate concentrations (˃11 mg l^−1^). Since November 2016, the water has stopped flowing and the values of parameters (mainly chlorides, sodium and calcium ions and conductivity) have risen drastically, while the values of biogens (nitrates and phosphates) have fallen below the level of determination. However, the value of chemical parameters measured in March 2017 was significantly lower (Table 1).

**Table 1:** Chemical parameters of water from the puddle in Stalowa Wola city

Sampling date	June 2016	November 2016	January 2017	March 2017
pH	9.4	7.9	7.9	7.8
Conductivity [μS cm^−1^]	347	13,250	22,100	3890
Cl^−^ [mg l^−1^]	64.56	4455.45	6195.00	40.83
SO_4_^2−^ [mg l^−1^]	74.02	21.35	36.44	14.82
NO_3_^−^ [mg l^−1^]	11.57	<0.001	<0.001	<0.001
PO_4_^3−^ [mg l^−1^]	<0.001	<0.001	<0.001	<0.001
NH_4_^+^ [mg l^−1^]	0.29	<0.001	<0.001	0.05
Na^+^ [mg l^−1^]	43.04	2689.24	3789.1	48.23
K^+^ [mg l^−1^]	24.43	66.72	46.65	3.50
Mg^2+^ [mg l^−1^]	8.35	38.48	21.98	6.26
Ca^2+^ [mg l^−1^]	41.73	145.86	125.5	9.45

**Fig. 2:**
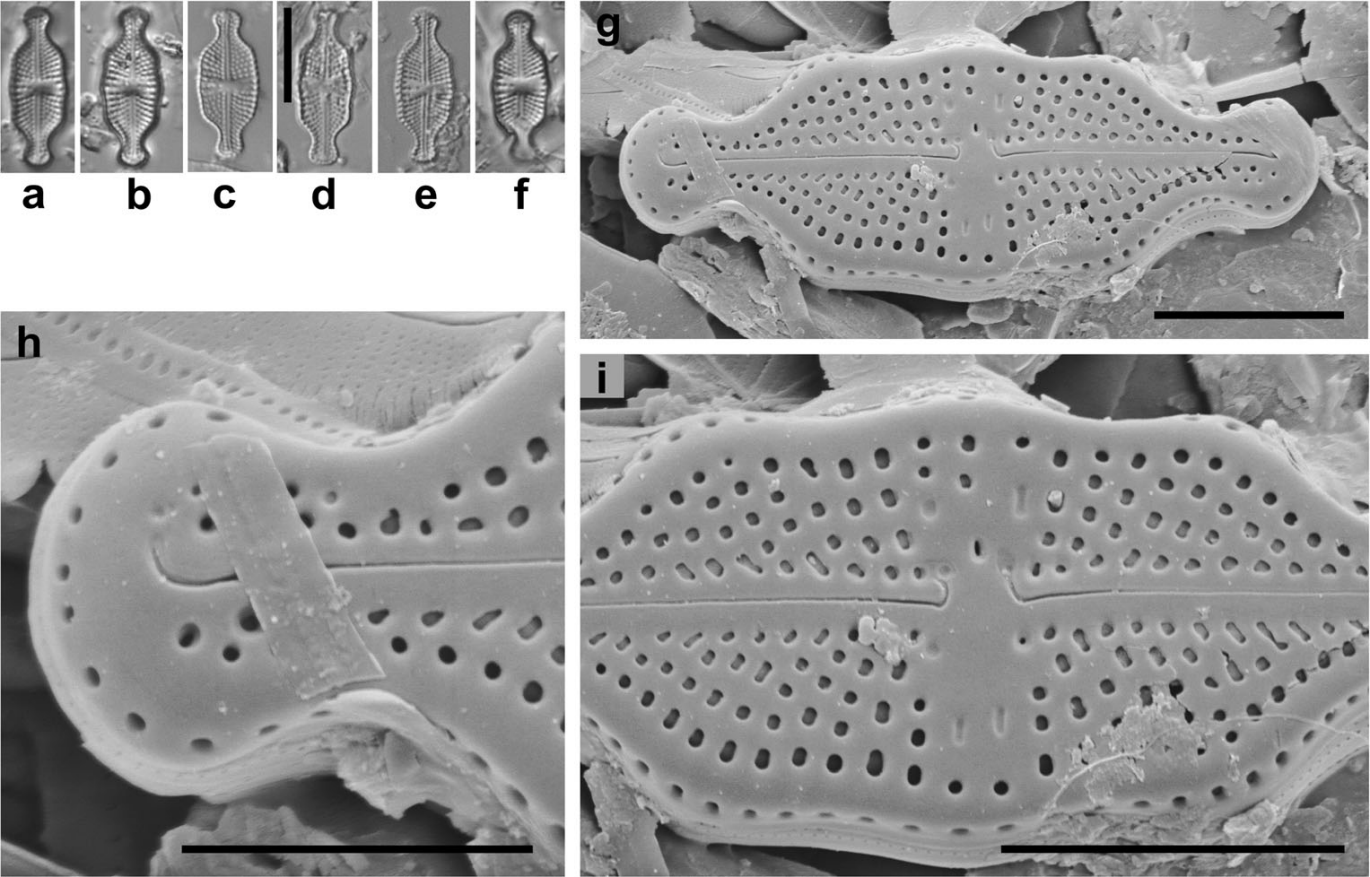
*Luticola vanheurckii* Van de Vijver & Levkov. **a**–**f** – LM photographs, scale bar = 10 μm. **g**–**i** – SEM external valve view, scale bar 5 = μm (**g**, **i**), 3 μm (**h**)

***Luticola vanheurckii*** Van de Vijver & Levkov (Fig. 2).

Valve description. Valves 14.9–18.7 μm long, 5.6–7.3 μm wide, striation density 18–21/10 μm, 3–5 areolae per striae. Observed in studied samples cells of *Luticola vanheurckii*, despite the larger size, correspond to the morphological characteristics included in the first species description by Levkov et al. (2013).

Distribution and ecology. The species occurred in a small puddle only rarely. This is the first record for Polish diatom flora, and the second for this species. Till now, *Luticola vanheurckii* was known only from the type locality – Groenedael, Belgium, but no information on its ecology and habitat was provided by Levkov et al. (2013). The species occurred in sediments and on plants submerged in water with a wide range of conductivity (347–22,100 μS cm^−1^), chloride content (64.56–6195.0 mg l^−1^) and sodium ions (43.04–3789.1 mg l^−1^).

In the assemblages with *L. vanheurckii* the most frequent diatom species were: *Nitzschia palea* (Kützing) W. Smith (45–74%), *Fistulifera saprophila* (Lange-Bertalot & Bonik) Lange-Bertalot (6–24%), *Gomphonema saprophilum* (Lange-Bertalot & Reichardt) Abraca, Jahn, Zimmermann & Enke (5–12%) and *Craticula minusculoides* (Hustedt) Lange-Bertalot (5–10%).

## Discussion

The habitat of *Luticola vanheurckii* in the city of Stalowa Wola was characterized by a very high electrolyte content (13250–22,100 μS cm^−1^), especially during the period when water stopped flowing. Very high concentrations of chlorides and sodium ions were measured mainly during the winter period, which could have been caused by sprinkling of road by salt (technical sodium chloride used in winter time).

The *L. vanheurckii* cells which were found in the examined material were slightly longer and wider (14.9–18.7 μm long and 5.6–7.3 μm wide vs. 12–18 μm long and 5–6,5 μm wide) in comparison to those described by Van de Vijver & Levkov (Levkov et al. 2013). Most of the valves observed in the study material had less striation (18–19/10 μm, only some 20–21 striae in 10 μm) than those given in an original description (Levkov et al. 2013).

*Luticola vanheurckii* may be confused with the following taxa: *L. pseudonivalis* (Bock) Levkov, Metzeltin & Pavlov, *L. pulchra* (McCall) Levkov, Metzeltin & Pavlov and *L. cocquytiae* Levkov, Metzeltin & Pavlov. Also, smaller specimens of *L. ventriconfusa* Lange-Bertalot and *L. undulata* (Hilse) D.G. Mann may be confused with *L. vanheurckii* (Levkov et al. 2013).

The most similar species to *L. vanheurckii* is *Luticola pseudonivalis* which coincides in dimension. *Luticola pseudonivalis* have a slightly more striae (22–24, not 18–22) and can be also distinguish by its more undulate margins. *Luticola pseudonivalis* have less constricted apices and proximal raphe endings deflected in opposite to the stigma, in contrast to *L. vanheurckii* in which proximal endings are deflected to stigma site. *Luticola pseudonivalis* is known only from light microscopy observations so detailed differences in valve structures between this taxa are not known. Previously *L. vanheurckii* could be identified as *L. pseudonivalis*. For example, some micrographs in Krammer and Lange-Bertalot (1986, pp. 563, Fig. 61:24) are similar to *L. vanheurckii*.

Another taxa with similar cell dimensions to *L. vanheurckii* are *L. pulchra* and *L. cocquytiae*, however, this species can be easily to distinguish by more undulate valves and by the shape of valve apices. Both species can be also differentiate from *L. vanheurckii* based on morphology of raphe endings. *Luticola vanheurckii* have distal and proximal raphe endings curved to stigma site while other two taxa have only distal raphe endings curved to site of stigma. In addiction distal raphe endings of *L. cocquytiae* are continuing onto valve mantle.

Another very similar to *L. vanheurckii* regarding its valve shape (mostly small valves) is *Luticola ventriconfusa*, which also have slightly undulate valves. Both taxa can be differentiate by less distinctly apices in *L. ventriconfusa*. Under SEM both taxa can be distinguish by distal raphe endings, which are continuing onto valve mantle in *L. ventriconfusa* while in *L. vanheurckii* distal raphe fissure are ends on valve face.

The most abundant species which were found in studied diatom assemblages with *L. vanheurckii* prefer eutrophic waters, with high or very high levels of electrolyte content, often polluted and with high saprobity (Lange-Bertalot 2001; Hofmann et al. 2011; Levkov et al. 2013). During our research, species growing in eutrophic waters with high saprobity (often found in polisaprobic waters) and which prefer brackish water were also identified: *Craticula buderii* (Hustedt) Lange-Bertalot, *C. cuspidata* (Kützing) D.G. Mann, *Navicula erifuga* Lange-Bertalot, *N. veneta* Kützing and *N. wiesneri* Lange-Bertalot (Lange-Bertalot 2001; Hofmann et al. 2011). In the diatom assemblages a lot of species from the genera *Mayamaea*, *Hantzschia*, *Luticola*, *Halamphora* and *Stauroneis* were identified, and which often develop on soils (Bąk et al. 2012; Hofmann et al. 2011; Stanek-Tarkowska and Noga 2012; Stanek-Tarkowska et al. 2013).

Till now, *Luticola vanheurckii* is known only from the type locality, Groenedael, Belgium. For this reason there is still a lack of information about the ecology of this species. Due to the presence of individual cells in sampling site in the Stalowa Wola, the ecological preferences of *L. vanheurckii* are still open.
